# Predictive Factors for Acute Postoperative Pain After Open Radical Gastrectomy for Gastric Cancer

**DOI:** 10.3389/fpubh.2022.907222

**Published:** 2022-06-01

**Authors:** Han Xie, Jingxuan Wei, Zhengliang Ma, Weihong Ge

**Affiliations:** ^1^Department of Pharmacy, Nanjing Drum Tower Hospital, The Affiliated Hospital of Nanjing University Medical School, Nanjing, China; ^2^State Key Laboratory of Quality Research in Chinese Medicines, Macau University of Science and Technology, Macau, Macau SAR, China; ^3^Department of Pharmacy, Nanjing Drum Tower Hospital Clinical College, Nanjing University of Chinese Medicine, Nanjing, China; ^4^Department of Anesthesiology, Nanjing Drum Tower Hospital, The Affiliated Hospital of Nanjing University Medical School, Nanjing, China

**Keywords:** gastric cancer, surgery, postoperative, acute pain, predictor

## Abstract

**Background:**

Pain has become an important factor in evaluating patients' quality of life and clinical treatment. For gastric cancer (GC) patients, open radical gastrectomy (OG) causes significant trauma to the body, increases patients' pain after operation, and delays early recovery. The aim of this study was to investigate the predictive factors of acute pain after OG within postoperative 72 h.

**Methods:**

From March 2020 to September 2021, 307 patients who underwent OG were included in the study in Nanjing Drum Tower Hospital. The predictors included demographic predictors, pathological data, surgical predictors, and intraoperative predictors. The pain scores at 12, 24, 48, and 72 h after operation were evaluated by numeric rating scale (NRS). The predictors of acute pain were determined by univariate and multivariate analysis.

**Results:**

The average pain score (NRS) of patients showed a downward trend over time within 72 h after OG. Multivariate analysis indicated that total gastrectomy (OR 1.823, 95% CI 1.094–3.040, *P* < 0.05), AJCC TNM stage (II) (OR.232, 95% CI 0.062–0.872, *P* < 0.05), AJCC TNM stage(III) (OR.185, 95% CI 0.049–0.698, *P* < 0.05), BMI (kg/m^2^) (OR 1.75, 95% CI 1.029–2.976, *P* < 0.05), distant metastasis (OR 3.054, 95% CI 1.019–9.155, *P* < 0.05), intraoperative transfusion (OR 2.246, 95% CI 1.267–3.982, *P* < 0.01) were significant predictive factors for acute pain after OG.

**Conclusion:**

Reasonable postoperative acute pain control was the prerequisite for accelerating the postoperative rehabilitation of patients. In order to reduce the occurrence of excessive or insufficient analgesia, it was necessary for patients who underwent OG to formulate appropriate analgesics according to risk factors.

## Introduction

Gastric cancer (GC) is a common malignant tumor of the digestive system, posing a significant risk to human health. According to global cancer statistics, GC has the fifth-highest incidence rate, and was the third leading cause of cancer deaths (1). The only hope for curing cancer stomach was radical gastrectomy (2). Depending on the tumor's location, it could remove all or part of the stomach. According to the classification of surgical methods, radical gastrectomy could be mainly divided into laparoscopic radical gastrectomy (LRG) and OG. LRG has developed rapidly since Kitano reported it for early GC in 1994 and has many advantages, including reducing bleeding, alleviating pain, and accelerating recovery (3–6). The therapeutic effect of LRG in patients with GC was increasingly prominent, especially for patients with early GC. The incidence of postoperative complications was lower, and the prognosis was better than OG (7, 8). However, for patients with advanced GC, clinical application's therapeutic effect and safety were still controversial. Moreover, surgery cost is relatively high because of high requirements for the technical level of equipment and physicians. The effectiveness and safety of LRG have also become the focus of clinicians and patients. Studies have shown that OG is safer when enlarged lymph nodes (ESLNs) are >2.5 cm (9). OG could effectively remove the lesions of patients and remove the surrounding lymph nodes as much as possible to improve the prognosis of patients and the survival rate of patients. However, it causes great trauma to the body, which increases the patients' pain invisibly. Moderate to severe postoperative acute pain could cause a strong stress response in patients, leading to decreased immune function, and a greater risk of postoperative tumor recurrence and metastasis, which directly and indirectly affects the prognosis (10).

Therefore, the study of factors affecting postoperative acute pain has important clinical significance for optimizing postoperative acute pain management (11). Doctors, nurses, and pharmacists need to understand the influencing factors of postoperative analgesic effect of the operation, intervene with these factors, and formulate individualized analgesic schemes, so as to reduce the occurrence of excessive or insufficient analgesia. In this article, 307 patients with GC after OG were followed up, and the factors that may affect the postoperative analgesic effect were analyzed, so as to provide reference for the formulation of postoperative analgesic scheme.

## Methods

### Patient and Public Involvement

This study was a retrospective single-center real-world study without any intervention in the treatment. This study was approved by the Ethics Committee of Nanjing Drum Tower Hospital, and the Ethics Committee agreed to waive the informed consent. GC patients who underwent OG at Nanjing Drum Tower Hospital from March 2020 to September 2021 were reviewed. Patients who met the following eligibility criteria were included: diagnosis of primary GC and accepted OG. All participants were Han Chinese. Patients with these conditions were excluded: remnant GC, history of other malignant tumors, quitting operation, and incomplete data.

### Perioperative Anesthesia and Surgical Procedure

All the research predictors were from patients who were anesthetized by the same team of anesthesiologists and operated by the same team of physicians. All patients underwent general anesthesia and OG.

Anesthesia information: All patients underwent total intravenous anesthesia. No premedication. The intravenous infusion pathway was established after the patient reached the operating room. Anesthesia was induced with midazolam (0.1 mg/kg), etomidate (0.2 mg/kg), cisatracurium besylate (0.4 mg/kg), and sufentanil (0.4 mg/kg). Target-controlled infusion (TCI) pump was used to maintain anesthesia with a target blood concentration of 4~6 mg/mL propofol; some patients were given patient-controlled intravenous analgesia (PCA) after surgery.

All patients underwent OG. The patients were placed in the supine position as the surgical position and subjected to general anesthesia. The abdominal region of the patients was routinely disinfected. The 15–20 cm around the navel in the middle of the upper abdomen was taken as the surgical incision. The subcutaneous tissue of the patients was stripped layer by layer to expose the lesions. The anatomical position of the organs in the abdominal cavity was carefully explored. The ultrasonic knife was used to complete the operation of gastric dissociation. The operator should strictly abide by the principle of tumor-free operation. At the same time, the corresponding lymph tissue should be cleaned according to the specific position of the tumor tissue. After the operation, the bleeding was completely stopped, and the abdominal cavity was thoroughly rinsed with sterile distilled water. The incision was sutured after the operation and covered with sterile dressing. Finally, the drainage tube was placed on the abdominal wall.

### Postoperative Analgesia

Postoperative patients received standard postoperative analgesia. PCA was given 10 min before the end of the operation. Fentanyl (adult: 15–20 mg/kg) was continuously infused, dexamethasone 10 mg, ondansetron 8 mg, diluted with normal saline, and the total volume was 100 ml. Dexamethasone and ondansetron prevent nausea or vomiting. The program was used for continuous infusion of background speed of 2 mL/h, a bolus dose of 0.5 mL, and lock for 15 min. Flurbiprofen axetil (50 mg b.i.d), parecoxib (40 mg b.i.d), or dezocine (10 mg b.i.d) as analgesics alleviate inflammation. If the patient complained of unbearable pain, intravenous pethidine was used as a rescue analgesic needed.

### Pain Intensity Measurement

Pain monitoring during hospitalization. The measurements were assessed using the American Society of Pain Guidelines for Postoperative Pain Management and the Chinese Society of Anesthesia Guidelines for Postoperative Pain Management. Pain measurement was performed at multiple time points (12, 24, 48, 72 h after operation) after the operation. The pain intensity was measured by NRS. NRS pain intensity score ranged from 0 to 10, 0 was painless, 10 was the most painful. Due to the implementation of postoperative acute pain management in our hospital, only 29.3% of patients after OG with NRS score ≥3 under the joint action of medical care and pharmacists. NRS = 3 as the cut-off value was not suitable for this study. Therefore, the NRS <2 was classified as a good analgesic effect (no pain), NRS ≥ 2 was classified as a poor analgesic effect (pain). Evaluating and recording NRS scores at multiple time points. Postoperative vomiting was recorded during follow-up. All the administrations were completed by the same postoperative acute pain management team composed of trained pharmacists.

### Predictors

The predictors included demographic predictors, pathological data, surgical predictors, and intraoperative predictors. We collected the participants' age, gender, BMI, diabetes, hypertension, previous abdominal surgery, pre-operative hemoglobin (g/L), pre-operative albumin (g/L), carcinoembryonic antigen, and pre-operative chemo- or radio-therapy before operation. We also recorded intraoperative information, such as American Society of Anesthesiologists physical status (ASA) score, total gastrectomy, or not intraoperative blood loss (ml), intraoperative fentanyl dosage (mg), intraoperative dexmedetomidine dosage (mg), and duration of operation (min). According to postoperative pathological data, we recorded tumor location, tumor size (cm), Lauren's histology, pathological grading, lymph node metastasis, depth of invasion, distant metastasis, lymphovascular invasion, and perineural invasion. Pathologic staging was evaluated according to the 8th American Joint Committee on Cancer (AJCC) staging system of GC.

### Statistics Analysis

IBM SPSS Statistics software (version 25.0; Chicago, IL) was used for statistical analysis. All continuous predictors were expressed by mean ± SD or median and quartiles (25th, 75th). All classification predictors were represented by percentages.

According to the distribution characteristics of data, Student *t* test or Mann- Whitney *U* test was used for univariate analysis to evaluate the related factors of patients. Categorical predictors were analyzed using the chi-squared test. In order to determine the risk factors for predicting poor analgesic effect, binary logistic regression was performed for multivariate analysis. Values of *P* < 0.05 were considered statistically significant.

## Results

**Figure 1:**
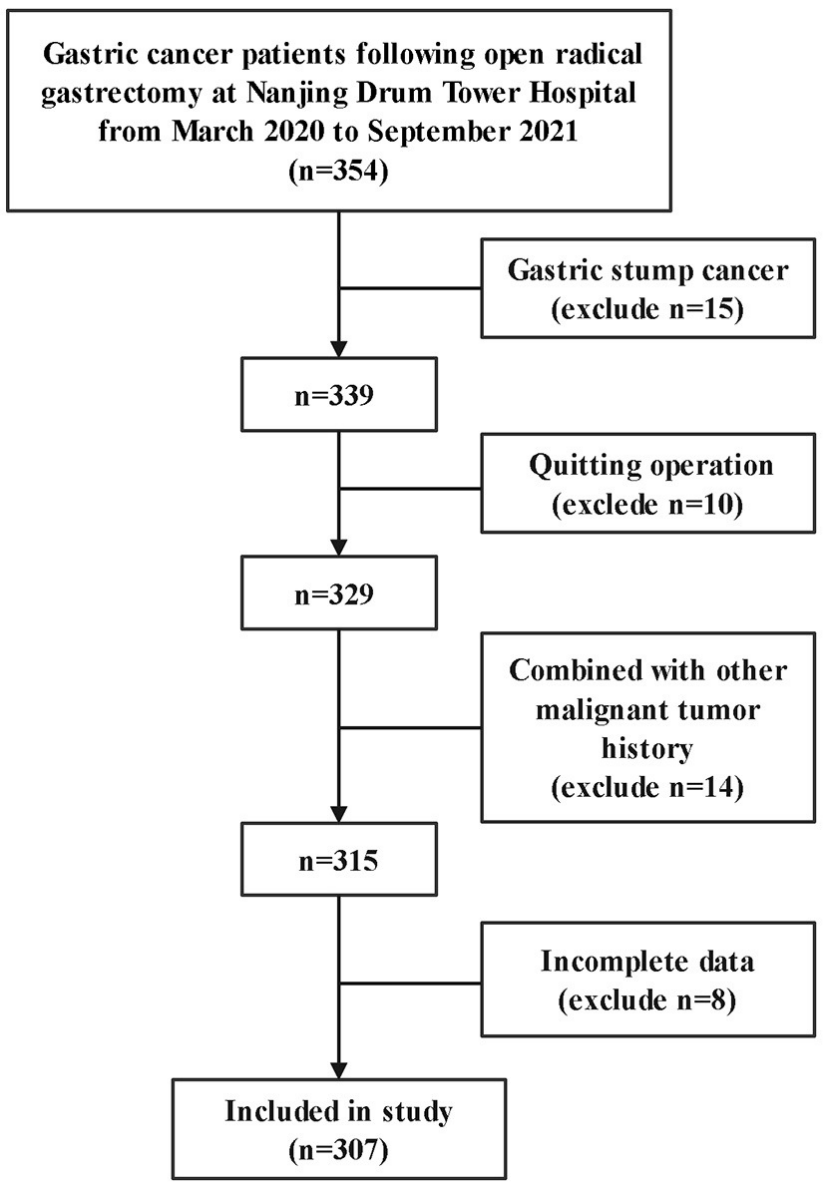
Research flowchart. A total of 307 patients were included in this study.

A total of 354 patients were close to participate in this study. 15 patients with gastric stump cancer, 10 patients who abandoned surgery, 14 patients with other malignant tumor histories, and 8 patients who had incomplete data were excluded from the study. Therefore, 307 patients were available for analysis (Figure 1).

### Descriptive Statistics

**Figure 2:**
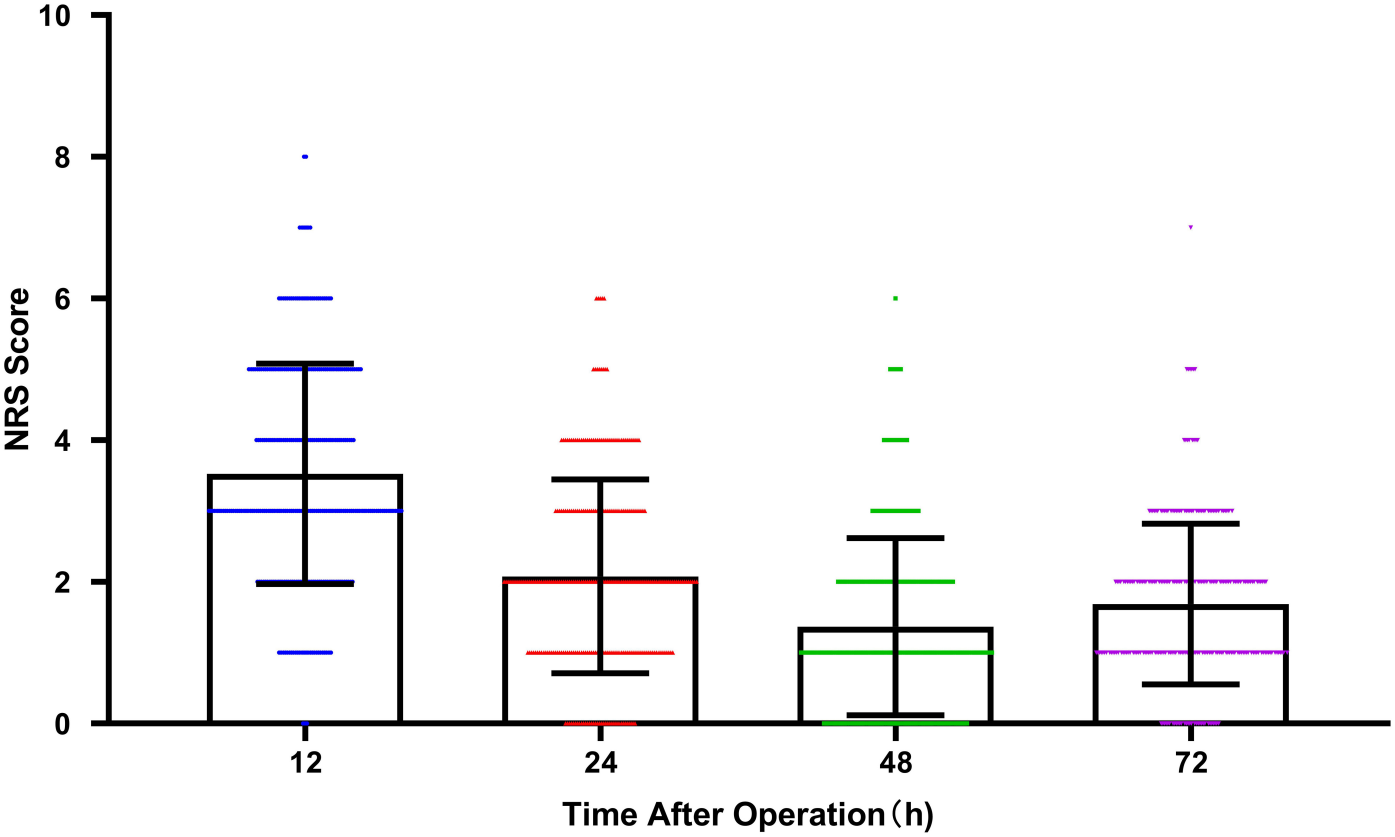
The average pain score (NRS) with time after operation at 12, 24, 48, and 72 h. Scatter plot with bar. The plot represented mean with SD. Color symbols represented individual values. (NRS, Numerical Rating Scale).

Demographics information, underlying diseases, data on surgery, and ASA classification were collected by researchers. Descriptive statistics for the patient characteristics are presented in Table 1. The mean patient age was 76.97 ± 9.80 years old, and 70 of the patients (22.8%) were female; 183 (59.6%) GC patients received total gastrectomy; 117 (38.1%) patients had hypertension; 46 (15.0%) patients had diabetes. Within 72 h after operation, a total of 197 (64.3%) patients suffered pain (NRS ≥ 2). PCA was provided for 21 (6.8%) patients for postoperative analgesia. For all patients, the average pain score changes at 24, 48, and 72 h after the operation are shown in Figure 2.

**Table 1:** Patient characteristics.

		**Postoperative NRS at 24 h**		**Postoperative NRS at 48 h**		**Postoperative NRS at 72 h**	
Predictors	Results (*n =* 307)	NRS <2	NRS ≥ 2	NRS <2	NRS ≥ 2	NRS <2	NRS ≥ 2
Number of scores recorded		110 (35.8%)	197 (64.3%)	191 (62.2%)	116 (37.8%)	151 (49.2%)	156 (50.8%)
Age, years	65.97 ± 9.80	66.69 ± 9.25	65.57 ± 10.09	66.33 ± 9.64	65.39 ± 10.06	66.03 ± 9.98	65.92 ± 9.66
**Gender**, ***n*** **(%)**							
Female	70 (22.8%)	20 (18.2%)	50 (25.4%)	46 (24.1%)	24 (20.7%)	30 (19.9%)	40 (25.6%)
Male	237 (77.2%)	90 (81.8%)	147 (74.6%)	145 (75.9%)	92 (79.3%)	121 (80.1%)	116 (74.4%)
**BMI, kg/m** ^**2**^							
<21	80 (26.1%)	29 (26.2%)	51 (25.9%)	40 (20.9%)	40 (34.5%)	31 (20.5%)	49 (31.4%)
≥21	227 (73.9%)	81 (73.6%)	146 (74.1%)	151 (79.1%)	76 (65.5%)	120 (79.5%)	107 (47.1%)
**Diabetes**							
No	261 (85.0%)	90 (81.8%)	171 (86.8%)	155 (81.2%)	106 (91.4%)	122 (80.8%)	139 (89.1%)
Yes	46 (15.0%)	20 (18.2%)	26 (13.2%)	36 (18.8%)	10 (8.6%)	29 (19.2%)	17 (10.9%)
**Hypertension**							
No	190 (61.9%)	68 (61.8%)	122 (61.9%)	116 (60.7%)	74 (63.8%)	85 (56.3%)	105 (67.3%)
Yes	117 (38.1%)	42 (38.2%)	75 (38.1%)	75 (39.3%)	42 (36.2%)	66 (43.7%)	51 (32.7%)
**Previous abdominal surgery**							
No	233 (72.6%)	80 (72.7%)	143 (72.6%)	136 (71.2%)	87 (75.0%)	113 (74.8%)	110 (70.5%)
Yes	84 (27.4%)	30 (27.3%)	54 (27.4%)	55 (28.8%)	29 (25.0%)	38 (25.2%)	46 (29.5%)
**Pre-operative hemoglobin, g/L**							
<120	166 (54.1%)	62 (56.4%)	104 (52.8%)	106 (55.5%)	60 (51.7%)	92 (60.9%)	74 (47.4%)
≥120	141 (45.9%)	48 (43.6%)	93 (47.2%)	85 (44.5%)	56 (48.3%)	59 (39.1%)	82 (52.6%)
**Pre-operative albumin, g/L**							
<35	46 (15.0%)	16 (14.5%)	30 (15.2%)	28 (14.7%)	18 (15.5%)	21 (13.9%)	25 (16.0%)
≥35	261 (85.0%)	94 (85.5%)	167 (84.8%)	163 (85.3%)	98 (84.5%)	130 (86.1%)	131 (84.0%)
**Carcinoembryonic antigen**							
<0.5	68 (22.1%)	22 (20.0%)	46 (23.4%)	43 (22.5%)	25 (21.6%)	38 (25.2%)	30 (19.2%)
0.5–10	214 (69.7%)	77 (70.0%)	137 (69.5%)	130 (68.1%)	84 (72.4%)	98 (64.9%)	116 (74.4%)
>10	25 (8.1%)	11 (10.0%)	14 (7.1%)	18 (9.4%)	7 (6.0%)	15 (9.9%)	10 (6.4%)
**Pre-operative chemo- or radio-therapy**							
No	294 (95.8%)	105 (95.5%)	189 (95.9%)	182 (95.3%)	112 (96.6%)	147 (97.4%)	147 (94.2%)
Yes	13 (4.2%)	5 (4.5%)	8 (4.1%)	9 (4.7%)	4 (3.4%)	4 (2.6%)	9 (5.8%)
**Tumor location**							
Upper 1/3	127 (41.4%)	37 (33.6%)	90 (45.7%)	80 (41.9%)	47 (40.5%)	65 (43.0%)	62 (39.7%)
Middle 1/3	72 (23.5%)	31 (28.2%)	41 (20.8%)	50 (26.2%)	22 (19.0%)	37 (24.5%)	35 (22.4%)
Lower 1/3	87 (28.3%)	35 (31.8%)	52 (26.4%)	48 (25.1%)	39 (33.6%)	40 (26.5%)	47 (30.1%)
2/3 or more	21 (6.8%)	7 (6.4%)	14 (7.1%)	13 (6.8%)	8 (6.9%)	9 (6.0%)	12 (7.7%)
**Tumor size (cm)**							
<3	79 (25.7%)	34 (30.9%)	45 (22.8%)	54 (28.3%)	25 (21.6%)	37 (24.5%)	42 (26.9%)
3–6	149 (48.5%)	51 (46.4%)	98 (49.7%)	92 (48.2%)	57 (49.1%)	76 (50.3%)	73 (46.8%)
>6	79 (25.7%)	25 (22.7%)	54 (27.4%)	45 (23.6%)	34 (29.3%)	38 (25.2%)	41 (26.3%)
**Lauren's histology**							
Intestinal type	152 (49.5%)	104 (68.9%)	109 (69.9%)	65 (59.1%)	87 (44.2%)	101 (52.9%)	51 (44.0%)
Diffuse type	58 (18.9%)	39 (25.8%)	43 (27.6%)	18 (16.4%)	40 (20.3%)	30 (15.7%)	28 (24.1%)
Mixed type	97 (31.6%)	8 (5.3%)	4 (2.6%)	27 (24.5%)	70 (35.5%)	60 (31.4%)	37 (31.9%)
**Pathological grading**							
Poorly differentiated	124 (40.4%)	42 (38.2%)	82 (41.6%)	74 (38.7%)	50 (43.1%)	59 (39.1%)	65 (41.7%)
Moderate differentiated	161 (52.4%)	60 (54.4%)	101 (51.3%)	103 (53.9%)	58 (50.0%)	80 (53.0%)	81 (51.9%)
Well differentiated	22 (7.2%)	8 (7.3%)	14 (7.1%)	14 (7.3%)	8 (6.9%)	12 (7.9%)	10 (6.4%)
**Lymph node metastasis**							
N0	116 (37.8%)	44 (40.0%)	72 (36.5%)	74 (38.7%)	42 (36.2%)	55 (36.4%)	61 (39.1%)
N1	40 (13.0%)	17 (15.5%)	23 (11.7%)	25 (13.1%)	15 (12.9%)	21 (13.9%)	19 (12.2%)
N2	59 (19.2%)	17 (15.5%)	42 (21.3%)	35 (18.3%)	24 (20.7%)	30 (19.9%)	29 (18.6%)
N3	92 (30.0%)	32 (29.1%)	60 (30.5%)	57 (29.8%)	35 (30.2%)	45 (29.8%)	47 (30.1%)
**Depth of invasion**							
T1–2	102 (33.2%)	39 (35.5%)	63 (32.0%)	62 (32.5%)	40 (34.5%)	45 (29.8%)	57 (36.5%)
T3–4	205 (66.8%)	71 (64.5%)	134 (68.0%)	129 (67.5%)	76 (65.5%)	106 (70.2%)	99 (63.5%)
**Distant metastasis**							
No	290 (94.5%)	107 (97.3%)	183 (92.9%)	182 (95.3%)	108 (93.1%)	139 (92.1%)	151 (96.8%)
Yes	17 (5.5%)	3 (2.7%)	14 (7.1%)	9 (4.7%)	8 (6.9%)	12 (7.9%)	5 (3.2%)
**Lymphovascular invasion**							
No	169 (55.0%)	58 (52.7%)	111 (56.3%)	106 (55.5%)	63 (54.3%)	84 (55.6%)	85 (54.5%)
Yes	138 (45.0%)	52 (47.3%)	86 (43.7%)	85 (44.5%)	53 (45.7%)	67 (44.4%)	71 (45.5%)
**Perineural invasion**							
No	144 (46.9%)	58 (52.7%)	86 (43.7%)	92 (48.2%)	52 (44.8%)	72 (47.7%)	72 (46.2%)
Yes	163 (53.1%)	52 (47.3%)	111 (56.3%)	99 (51.8%)	64 (55.2%)	79 (52.3%)	84 (53.8%)
**AJCC TNM stage**							
I	83 (27.0%)	32 (29.1%)	51 (25.9%)	53 (27.7%)	30 (25.9%)	36 (23.8%)	47 (30.1%)
II	65 (21.2%)	31 (28.2%)	34 (17.3%)	43 (22.5%)	22 (19.0%)	35 (23.2%)	30 (19.2%)
III	139 (45.3%)	44 (40.0%)	95 (48.2%)	83 (43.5%)	56 (48.3%)	70 (46.4%)	69 (44.2%)
IV	20 (6.5%)	3 (2.7%)	17 (8.6%)	12 (6.3%)	8 (6.9%)	10 (6.6%)	10 (6.4%)
**ASA score**							
II	20 (6.5%)	6 (5.5%)	14 (7.1%)	11 (5.8%)	9 (7.8%)	11 (7.3%)	9 (5.8%)
III	251 (81.8%)	91 (82.7%)	160 (81.2%)	156 (81.7%)	95 (81.9%)	121 (80.1%)	130 (83.3%)
IV	35 (11.4%)	13 (11.8%)	22 (11.2%)	24 (12.6%)	11 (9.5%)	18 (11.9%)	17 (10.9%)
V	1 (0.3%)	0 (0.0%)	1 (0.5%)	0 (0%)	1 (0.9%)	1 (0.7%)	0 (0%)
**Total gastrectomy**							
No	124 (40.4%)	36 (32.7%)	88 (44.7%)	76 (39.8%)	48 (41.4%)	57 (37.7%)	67 (42.9%)
Yes	183 (59.6%)	74 (67.3%)	109 (55.3%)	115 (60.2%)	68 (58.6%)	94 (62.3%)	89 (57.1%)
**Intraoperative blood loss, ml**							
<100	15 (4.9%)	8 (7.3%)	7 (3.6%)	13 (6.8%)	2 (1.7%)	8 (5.3%)	7 (4.5%)
≥100	292 (95.1%)	102 (92.7%)	190 (96.4%)	178 (93.2%)	114 (98.3%)	143 (94.7%)	149 (95.5%)
**Intraoperative transfusion, ml**							
<100	240 (78.2%)	82 (74.5%)	158 (80.2%)	147 (77.0%)	93 (80.2%)	108 (71.5%)	132 (84.6%)
≥100	67 (21.8%)	28 (25.5%)	39 (19.8%)	44 (23.0%)	23 (19.8%)	43 (28.5%)	24 (15.4%)
Intraoperative fentanyl dosage, mg	0.63 ± 0.22	0.64 ± 0.21	0.62 ± 0.23	0.64 ± 0.21	0.60 ± 0.25	0.64 ± 0.21	0.61 ± 0.24
Intraoperative dexmedetomidine dosage, mg	38.47 ± 18.95	38.25 ± 21.71	38.59 ± 17.29	38.61 ± 18.97	38.23 ± 19.02	39.59 ± 17.17	37.39 ± 20.53
**Duration of operation, min**							
<180	92 (30.0%)	31 (28.2%)	61 (66.3%)	49 (25.7%)	43 (37.1%)	51 (33.8%)	41 (26.3%)
≥180	215 (70.0%)	79 (71.8%)	136 (44.3%)	142 (74.3%)	73 (62.9%)	100 (66.2%)	115 (73.7%)
**Postoperative PCA**							
No	286 (93.2%)	104 (94.5%)	182 (92.4%)	176 (92.1%)	110 (94.8%)	139 (92.1%)	147 (94.2%)
Yes	21 (6.8%)	6 (5.5%)	15 (4.9%)	15 (7.9%)	6 (5.2%)	12 (7.9%)	9 (5.8%)
**Preventive analgesia**							
No preventive analgesia	9 (2.9%)	4 (3.6%)	5 (2.5%)	4 (2.1%)	5 (4.3%)	4 (2.6%)	5 (3.2%)
Flurbiprofen axetil (50 mg b.i.d)	123 (40.1%)	38 (34.5%)	85 (43.1%)	77 (40.3%)	46 (39.7%)	61 (40.4%)	62 (39.7%)
Parecixib (40 mg b.i.d)	29 (9.4%)	13 (11.8%)	16 (8.1%)	21 (11.0%)	8 (6.9%)	13 (8.6%)	16 (10.3%)
Dezocine (10 mg b.i.d)	146 (47.6%)	55 (50.0%)	91 (46.2%)	89 (46.6%)	57 (49.1%)	73 (48.3%)	73 (46.8%)

*Predictors are shown as mean ± SD, median with median (25th, 75th) when appropriate.*

*ASA Classification, American Society of Anesthesiologists physical status; BMI, body mass index; NRS, Numerical Rating Scale; AJCC, American Joint Committee on Cancer; TNM, Tumor Node Metastasis; PCA, Patient-controlled intravenous analgesia*.

### Univariate Analysis

Our study assessed the pain scores at 24, 48, and 72 h after surgery. Table 2 showed the data analysis results. At postoperative 24 h, whether total gastrectomy was performed or not (*P* < 0.05), and AJCC TNM stage (*P* < 0.05) was related to postoperative acute pain after OG. At postoperative 48 h, BMI (*P* < 0.01), diabetes (*P* < 0.05), hypertension (*P* < 0.05), Lauren's histology (*P* < 0.05), intraoperative blood loss (*P* < 0.05), and duration of operation (*P* < 0.05) were related to postoperative acute pain. At postoperative 72 h, BMI (*P* < 0.05), diabetes (*P* < 0.05), pre-operative hemoglobin (*P* < 0.05), intraoperative blood transfusion (*P* < 0.01) were related to postoperative acute pain. BMI and diabetes were both associated with postoperative acute pain at 48 and 72 h. In addition, there was a difference in the patient sources between groups, but this difference did not reach statistical significance. We used these predictors in the multivariate analysis.

**Table 2:** Univariate analysis of predictive factors for pain within 72 h after OG.

	**Postoperative NRS at 24 h**	**Postoperative NRS at 48 h**	**Postoperative NRS at 72 h**
**Predictors**	***P value***	***P value***	***P value***
Age, years	0.308	0.509	0.917
Gender, *n* (%)	0.149	0.492	0.228
BMI, kg/m^2^	0.928	0.009*	0.03*
Diabetes	0.241	0.015*	0.041*
Hypertension	0.985	0.592	0.047*
Previous abdominal surgery	0.979	0.469	0.396
Pre-operative hemoglobin, g/L	0.547	0.520	0.018*
Pre-operative albumin, g/L	0.872	0.838	0.603
Carcinoembryonic antigen	0.581	0.537	0.185
Pre-operative chemo- or radio-therapy	0.841	0.594	0.175
Tumor location	0.179	0.325	0.792
Tumor size, cm	0.279	0.332	0.815
Lauren's histology	0.457	0.040*	0.144
Pathological grading	0.838	0.752	0.820
Lymph node metastasis	0.512	0.953	0.942
Depth of invasion	0.535	0.715	0.210
Distant metastasis	0.108	0.417	0.049*
Lymphovascular invasion	0.541	0.839	0.841
Perineural invasion	0.127	0.570	0.789
AJCC TNM stage	0.028*	0.817	0.622
ASA score	0.744	0.392	0.603
Total gastrectomy	0.041*	0.783	0.353
Duration of operation, min	0.610	0.034*	0.152
Intraoperative blood loss, ml	0.147	0.045*	0.742
Intraoperative transfusion, ml	0.250	0.509	0.005*
Postoperative PCA	0.472	0.367	0.450
Preventive analgesia	0.419	0.458	0.951
Intraoperative fentanyl dosage, mg	0.280	0.860	0.288
Intraoperative dexmedetomidine dosage, mg	0.593	0.865	0.311

*ASA Classification, American Society of Anesthesiologists physical status; BMI, body mass index; NRS, Numerical Rating Scale; AJCC, American Joint Committee on Cancer; TNM, Tumor Node Metastasis; PCA, Patient-controlled intravenous analgesia.*

*P < 0.05.

### Multivariate Analysis

**Figure 3:**
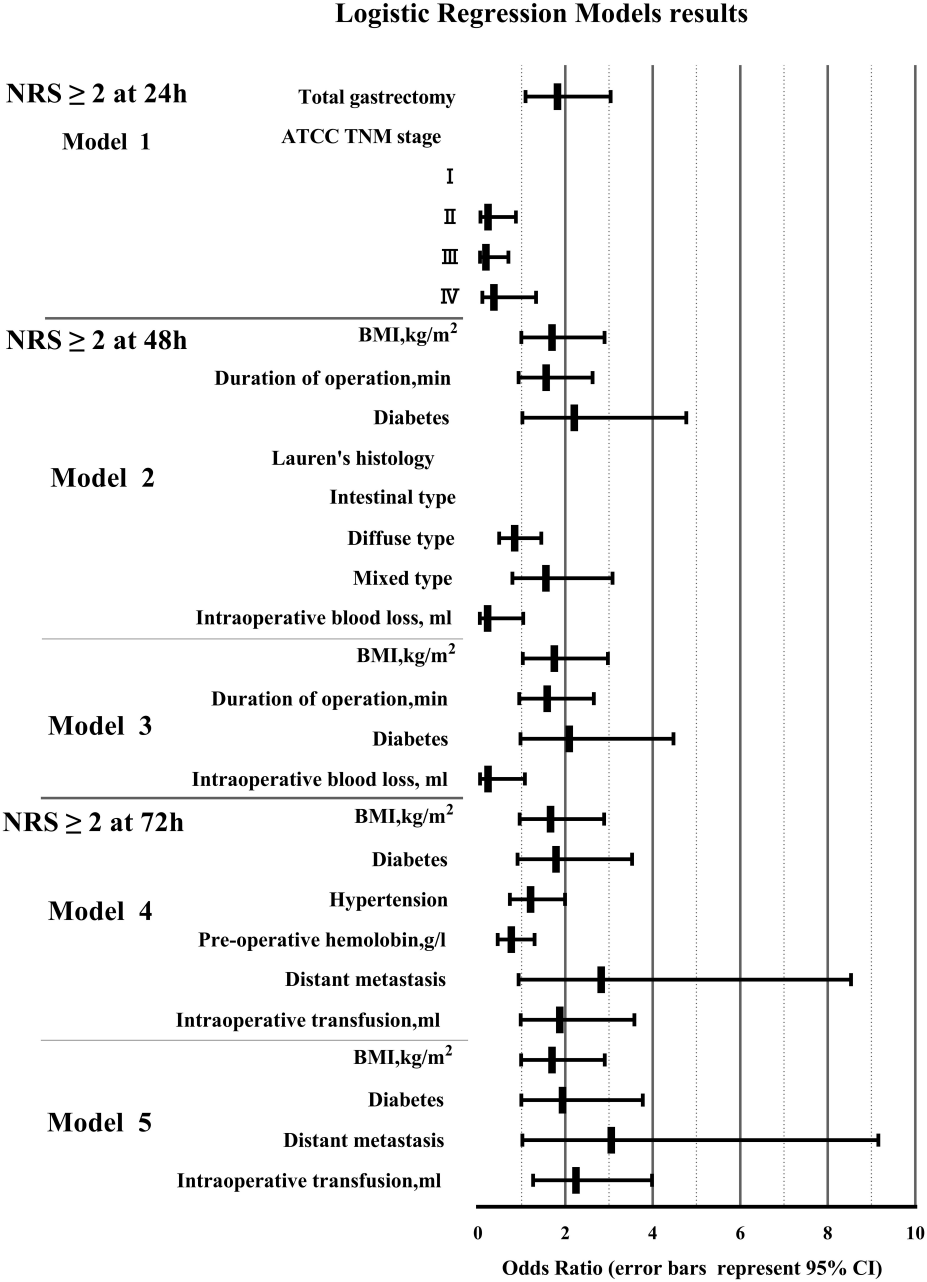
Binary logistic regression model results. (BMI, body mass index; NRS, Numerical Rating Scale; AJCC, American Joint Committee on Cancer; TNM, Tumor Node Metastasis).

To determine the risk factors of pain after OG, binary logistic regression was used to investigate the predictors that showed a significant difference (*P* < 0.05) in the univariate analysis (Table 3 and Figure 3). After 24 h post-operation, the significant predictors included total gastrectomy (OR 1.823, 95% CI 1.094–3.040, *P* < 0.05), AJCC TNM stage (II) (OR 0.232, 95% CI 0.062–0.872, *P* < 0.05), and AJCC TNM stage (III) (OR 0.185, 95% CI 0.049–0.698, *P* < 0.05). After operation 48 h, the significant predictors included BMI (kg/m^2^) (OR 1.75, 95% CI 1.029–2.976, *P* < 0.05). After operation 72 h, the significant predictors included distant metastasis (OR 3.054, 95% CI 1.019–9.155, *P* < 0.05), intraoperative transfusion (OR 2.246, 95% CI 1.267–3.982, *P* < 0.01).

**Table 3:** Binary logistic regression analysis for outcome postoperative NRS at 24, 48, 72 h.

	**Outcome: NRS ≥2 at postoperative 24 h**			
**Predictors**		**Model 1**		
		**OR (95% CI)**	***P value***	
Total gastrectomy		1.823 (1.094–3.040)	0.021*	
AJCC TNM stage				
I	(reference)			
II		0.232 (0.062–0.872)	0.031*	
III		0.185 (0.049–0.698)	0.013*	
IV		0.369 (0.102–1.332)	0.128	
	**Outcome: NRS** **≥2 at postoperative 48 h**			
Predictors	**Model 2**		**Model 3**	
	**OR (95% CI)**	***P***	**OR (95% CI)**	***P***
BMI, kg/m^2^	1.699 (0.995–2.900)	0.052	1.75 (1.029–2.976)	0.039*
Duration of operation, min	1.565 (0.933–2.625)	0.090	1.587 (0.95–2.652)	0.078
Diabetes	2.205 (1.02–4.765)	0.044*	2.09 (0.977–4.473)	0.057
**Lauren's histology**				
Intestinal type	(reference)			
Diffuse type	0.841 (0.487–1.454)	0.536		
Mixed type	1.558 (0.788–3.081)	0.202		
Intraoperative blood loss, ml	0.223 (0.048–1.042)	0.056	0.234 (0.051–1.076)	0.062
	**Outcome: NRS** **≥2 at postoperative 72 h**			
Predictors	**Model 4**		**Model 5**	
	**OR (95% CI)**	***P***	**OR (95% CI)**	***P***
BMI, kg/m^2^	1.663 (0.957–2.890)	0.071	1.697 (0.992–2.905)	0.054
Diabetes	1.791 (0.909–3.528)	0.092	1.939 (0.997–3.771)	0.051
Hypertension	1.209 (0.732–1.996)	0.459		
Pre-operative hemoglobin, g/L	0.767 (0.454–1.297)	0.322		
Distant metastasis	2.821 (0.932–8.535)	0.066	3.054 (1.019–9.155)	0.046*
Intraoperative transfusion, ml	1.876 (0.983–3.581)	0.056	2.246 (1.267–3.982)	0.006*

*BMI, body mass index; NRS, Numerical Rating Scale; AJCC, American Joint Committee on Cancer; TNM, Tumor Node Metastasis.*

*P <0.05.

*Explanation for models Binary logistic regression models 1,2,4 were constructed using predictors found to be significant in the univariate analysis (p <0.05). Models 3,5 were derived from models 2,4 respectively with non-significant predictors eliminated in stepwise process called backward conditional. The resulting models include only significant predictors (p <0.05). The reported odds ratios (all significant ones are above 1) suggest that one unit increase in predictor score (or having categorical predictor) is associated with increase odds of pain*.

## Discussion

As one of the most common malignant tumors of the digestive system, GC posed a serious threat to people's lives and health (12). The results of this study showed that the pain scores of patients showed a downward trend over time within 72 h after surgery. However, the pain score at 72 h was slightly higher than that at 48 h, which may be related to wound dressing change and drainage tube removal. Some patients had a tolerance to analgesics, and the withdrawal of PCA (48–72 h after surgery).

To determine independent predictors of pain after OG within 72 h, we used binary logistic regression models after univariate analysis. There were so many variables included in this study, including demographics information, pathological data, and surgical data. Univariate analysis was carried out to screen out some variables which may be meaningful. And then binary logistic regression analysis was performed on variables with differences (*P* < 0.1). Binary logistic regression analysis used backward conditional, eliminated non-local variables step by step, and finally got 5 significant predictive factors (*P* < 0.05). It could not only explain the correlation between variables and postoperative acute pain after OG, but also reflect the strength of the correlation through OR value. In this study, total gastrectomy, AJCC TNM stage (I), BMI≥21 kg/m^2^, distant metastasis, intraoperative blood transfusion (≥100 ml) were risk factors for postoperative acute pain.

In our study, total gastrectomy or proximal or distal gastrectomy was an important factor affecting postoperative acute pain. Total gastrectomy had potential advantages in improving the long-term survival rate and reducing the incidence of residual GC (13). Compared with proximal or distal gastrectomy, total gastrectomy had a longer operation time and more intraoperative blood loss. Activated injury receptors or immune cells released a large number of endogenous inflammatory mediators (14). At the same time, injury receptors expressed one or more cell surface receptors, such as G protein-coupled receptors (GPCR) and *N*-methyl-*D*-aspartic acid (NMDA). These receptors specifically recognized the corresponding inflammatory mediators, enhancing the excitability nerve fibers, and improving the sensitivity of injury receptors to injurious stimuli (15). Laparoscopic distal gastrectomy for TNM stage I-III GC had less blood loss, less postoperative pain, and mild inflammatory response (16).

We found that BMI correlated with postoperative acute pain (*P* = 0.039) after OG. Most studies from Asian Centers used BMI value of 25 kg/m^2^ as the critical value for dividing patients into obesity, which was inconsistent with the current definition of obesity by the WHO (17). In a meta-analysis, the effect of obesity on the prognosis of GC after resection was studied, and BMI ≥ 30 was defined as obesity (18). Intraoperative blood loss was reported in 4 studies and was lower in the non-obese group, but the difference was not statistically significant (19–22). Similarly, non-obese patients could be observed in wound infection decreased trend, but this did not reach the level of statistical significance (22). Excessive visceral fat wrapped in the main blood vessels of the upper abdomen may affect the recognition of the best anatomical plane, and the operation time may be longer. Increased blood loss, increased risk of wound infection, and prolonged operation time were potential factors for postoperative acute pain.

Our study suggested that patients at different TNM stages of cancer may respond differently to postoperative acute pain. A retrospective study investigated the effect of postoperative systemic inflammation on prognosis in patients with TNM stage I GC, and suggested that early postoperative serum C-reactive protein (CRP) level (cut-off value was 13.9 mg/dL) could predict the long-term prognosis of radical gastrectomy (23). Saito et al. evaluated the effect of CRP peak level on prognosis in patients with advanced GC after radical gastrectomy and identified CRP peak level (cut-off value was 12 mg/dL) as an independent prognostic factor (24). CRP is synthesized by the liver, mainly regulated by interleukin-6, and may upregulate pro-inflammatory and anti-inflammatory cytokines (25). Recently, some studies have shown that postoperative systemic inflammation is significantly correlated with the postoperative prognosis of cancer patients through evaluating serum CRP level (25–27). The increase of postoperative CRP level in patients with GC may predict the increase of inflammatory level, and strong inflammatory response may cause serious postoperative acute pain.

According to the 8th AJCC TNM classification system, no matter the depth of tumor penetrating the gastric wall (T) and the number and state of lymph nodes (N), distant metastasis is divided into stage IV. Patients at the IV stage usually suffer from a long and painful illness. Postoperative patients in our hospital would use non-steroidal anti-inflammatory drugs combined with opioids analgesia. Opioids play an analgesic effect by simulating the physiological role of endogenous opioid peptides (28). Patients with advanced GC faced low cholesterol levels due to malnutrition. Low cholesterol levels may reduce the activity of opioids (29). Studies have shown that patients with lung cancer at low cholesterol levels need higher doses of opioids to achieve the same level of pain control (30). Our study also confirmed that patients with distant metastasis were more likely suffer acute pain than patients with early GC after surgery.

In our study, blood transfusion was an independent predictor of postoperative acute pain. Blood transfusion could save a life in many cases but had a negative influence on immune regulation, postoperative infection, and tumor metastasis, and recurrence (31). Immunomodulation of the innate and adaptive immune system occurred after exposure of the recipient to the many cell-bound and soluble antigens which were expressed on viable and decaying cells in the transfusion (32). Blood transfusion was associated with infectious complications following gastrointestinal surgery (33). The activation of inflammation during blood transfusion was closely related to the severity of postoperative pain. A meta-analysis also confirmed that the restrictive allogeneic blood transfusion strategy could reduce the perioperative infection rate without increasing the incidence of complications such as cardiac events or mortality (34). Retrospective analysis of a single central database also confirmed that perioperative blood transfusion was independently associated with poor prognosis in patients with GC (35).

Our study also had some limitations. We only evaluated and explored the possible factors affecting pain within 72 h after surgery. There was no study on the influencing factors of pain 3 days and long-term after surgery. At the same time, our research was limited to OG, and there was no study on the influencing factors of pain after LRG and robotic radical gastrectomy for GC. In addition, postoperative acute pain was affected by genetic polymorphism related to pharmacokinetics, pharmacodynamics of analgesics (36) and psychology, and we had not studied these influencing factors.

Pain has become an important factor in evaluating patients' quality of life and clinical treatment. Medical staff should predict the influencing factors of postoperative acute pain, formulate reasonable analgesic schemes, and reduce the occurrence of excessive analgesia and insufficient analgesia. Reasonable postoperative pain control was the prerequisite for accelerating the postoperative rehabilitation of patients.

Total gastrectomy, AJCC TNM stage (I), BMI (≥21, kg/m^2^), distant metastasis, and intraoperative transfusion (≥100 ml) were significantly associated with pain after OG within postoperative 72 h. To reduce the occurrence of excessive analgesia and insufficient analgesia, formulating appropriate analgesics according to these risk factors was necessary for patients who underwent OG.

## Data Availability Statement

The original contributions presented in the study are included in the article/Supplementary Material, further inquiries can be directed to the corresponding authors.

## Ethics Statement

The studies involving human participants were reviewed and approved by Ethics Committee of Nanjing Drum Tower Hospital. Written informed consent for participation was not required for this study in accordance with the national legislation and the institutional requirements.

## Author Contributions

HX and JW: design. JW and HX: writing. ZM and JW: analysis. WG and MH: methodology. JW and WG: data curation. All authors read and approved the final manuscript.

## Funding

This work was supported by the National Natural Science Foundation of China (No. 72104105) and Special Fund for Clinical Research of Nanjing Drum Tower Hospital (No. 2021-LCYJ-PY-07). HX was responsible for the overall content of these funding as guarantor. The guarantor accepted full responsibility for the finished work and the conduct of the study, had access to the data, and controlled the decision to publish.

## Conflict of Interest

The authors declare that the research was conducted in the absence of any commercial or financial relationships that could be construed as a potential conflict of interest.

## Publisher's Note

All claims expressed in this article are solely those of the authors and do not necessarily represent those of their affiliated organizations, or those of the publisher, the editors and the reviewers. Any product that may be evaluated in this article, or claim that may be made by its manufacturer, is not guaranteed or endorsed by the publisher.

## Abbreviations

AJCC: American Joint Committee on Cancer
ASA: American Society of Anesthesiologists physical status
CRP: C-reactive protein
ESLNs: Enlarged lymph nodes
GC: Gastric cancer
GPCR: G protein-coupled receptors
LRG: Laparoscopic radical gastrectomy
NMDA: *N*-methyl-*D*-aspartic acid
NRS: Numerical rating scale
OG: Open radical gastrectomy
PCA: Patient-controlled intravenous analgesia
TCI: Target-controlled infusion
TNM: Tumor node metastasis
WHO: World health organization.
